# Portrait of replication stress viewed from telomeres

**DOI:** 10.1111/cas.12165

**Published:** 2013-05-12

**Authors:** Fuyuki Ishikawa

**Affiliations:** Graduate School of Biostudies, Kyoto UniversityKyoto, Japan

## Abstract

Genetic instability is the driving force of the malignant progression of cancer cells. Recently, replication stress has attracted much attention as a source of genetic instability that gives rise to an accumulation of mutations during the lifespan of individuals. However, the molecular details of the process are not fully understood. Here, recent progress in understanding how genetic alterations accumulate at telomeres will be reviewed. In particular, two aspects of telomere replication will be discussed in this context, covering conventional semi-conservative replication, and DNA synthesis by telomerase plus the C-strand fill-in reactions. Although these processes are seemingly telomere-specific, I will emphasize the possibility that the molecular understanding of the telomere events may shed light on genetic instability at other genetic loci in general.

Recent progress in cancer genome analysis, aided by rapidly advancing DNA sequencing technology, has confirmed and elaborated earlier, lower resolution cytogenic observations that cancer cells acquire a variety of structural changes of chromosomes. Specifically, it is important to reveal differential modes of chromosomal rearrangements happening at oncogene and tumor suppressor loci, and clues as to how such seemingly distinct chromosomal changes occur.^1^ It has been revealed that the timely progression of the numerous replication forks that copy the huge genomic DNA is often perturbed during each S phase.^2^

The telomere is a chromosomal domain essential for the faithful maintenance of the genome. It protects the end of a linear genomic DNA from illegitimate DNA repair reactions and prevents activation of the DNA damage checkpoint. It coordinates efficient telomere DNA replication by the conventional replication fork and telomerase. Recent studies have greatly advanced our understanding of how telomere defects lead to massive chromosomal instability. Here I will describe the molecular mechanism that ensures telomere integrity in the face of replication stress.

## Telomere Chromatin

**Figure 1:**
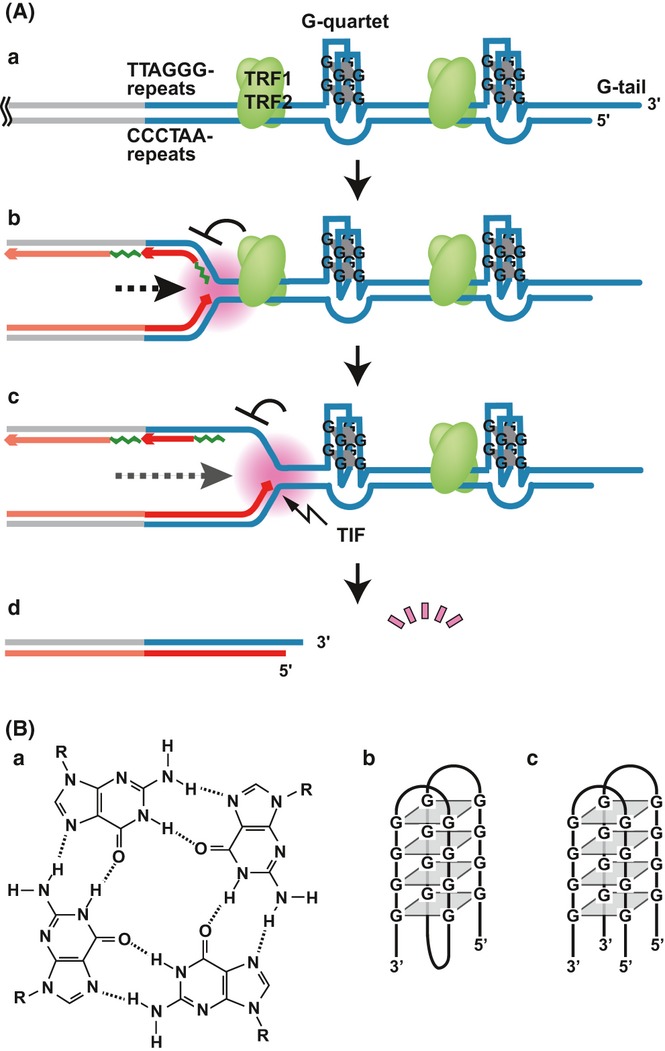
(A) Schematic representation of how telomere DNA is replicated. [a] Vertebrate telomere duplex DNA consists of G-rich and C-rich repetitive DNA strands (TTAGGG-repeats and CCCTAA-repeats, respectively). The G-strand DNA may form intra-molecular G-quartets as schematically indicated. The C-strand DNA unpaired with G-strands participating in G-quartet formation is supposed to be single-stranded. [b] When DNA replication fork moves distally at telomere regions, it encounters the higher-ordered structure of G-quartet, thereby reducing the rate of DNA synthesis. The spatial hindrance of telomere chromatin may block the replication fork movement. [c] The replication fork stalling may leave unreplicated ssDNA template, which activates the ATR intra-S phase checkpoint. Telomeres are recognized as DNA damages by cells. TIF, telomere dysfunction-induced focus. [d] The unreplicated ssDNA template may be eventually broken down to form DNA double-stranded breaks (DSBs). DSBs can trigger a variety of chromosomal rearrangements through the end-joining and homologous recombination pathways. Telomere DNA is massively shortened in a single step. Arrowed red and orange lines indicate nascent DNA strands. Wavy green lines indicate RNA primers. Black and gray arrows show the direction of replication fork movement. (B) Structures of Hoogsteen base-pairing and G-quartet [reviewed in^39^]. [a] Four guanine nucleotides form a planar association, which is called Hoogsteen base-pairing. [b,c] Four single-stranded DNAs containing stretches of consecutive guanines form the G-quartet structure. In this panel, four stretches of four guanines (G) form four layers of G-G Hoogsteen base-pairing (indicated by shaded squares). [b] and [c] show intra-molecular and intermolecular G-quartets, respectively.

Vertebrate telomere DNA consists of double-stranded (ds) TTAGGG/CCCTAA repeats. The 3′-end of TTAGGG-repeat DNA (called the G-strand) and the 5′-end of CCCTAA-repeat DNA (called the C-strand) are at the DNA termini (Fig. 1A). At the extreme end of the G-strand DNA is a single-stranded extension, called the G-tail. The length of human ss G-tail is approximately 50–200 nt. The length of the ds telomere repeats is variable among different species. Human cells typically show approximately from several kb to 20 kb. Notably, congenic mouse strains conventionally used in mouse genetics have larger telomere DNAs spanning from 30 to over 100 kb.^3^ This should be taken into account when results obtained from experiments using experimental mice are interpreted. Indeed, mouse telomere lengths were not noticeably reduced in the first generation of telomerase knockout mice. Only with the fourth generation and thereafter, phenotypes including chromosome instability and tissue atrophy were observed in the knockout mice.^4^

**Figure 2:**
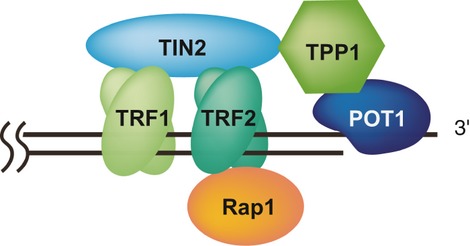
Vertebrate shelterin complex. TRF1 and TRF2 directly bind to ds telomere DNA. Pot1 bind to ss G-strand DNA (G-tail). TPP1, Rap1 and POT1 are recruited to telomeres by protein–protein interactions.

The vertebrate telomere DNA associates with both conventional nucleosomes,^5^ and non-histone proteins. The non-histone proteins are either telomere-specific or telomere-non-specific, and they either constitutively or cell-type/cell-cycle specifically associate with telomeres. A protein complex called shelterin forms the constitutive telomere architecture that is required for vital telomere function.^5^ Shelterin consists of six proteins, TRF1, TRF2, TIN2, Rap1, TPP1 and POT1 (Fig. 2). TRF1 and TRF2 directly bind ds telomere DNA, while POT1 binds ss telomere DNA. TRF1 negatively regulates telomerase-mediated telomere elongation, as evidenced by the fact that overexpression and knockdown of TRF1 resulted in telomere shortening and elongation, respectively.^6^

## Hard Life at Telomeres

The SV40 *in vitro* replication system recapitulates conventional semi-conservative DNA replication. Using this system, it was observed that the replication fork is frequently stalled at telomere repeat DNAs that were included in the template plasmid. Furthermore, it was suggested that the replication fork progressed slowly at telomeres in HeLa cells when TRF1 or TRF2 was overexpressed.^7^ These results suggested that TRF1 and/or TRF2-bound telomere chromatin was a poor substrate for DNA replication. The link between telomere chromatin and DNA replication was underscored when it was found that ds telomere DNA-binding protein Taz1 is required for efficient DNA replication at telomeres in fission yeast *Schizosaccharomyces pombe*.^8^–^9^ In line with these results, human TRF1 is required for the efficient semi-conservative replication of ds telomere DNA.^10^ When TRF1 was conditionally deleted, cells failed to replicate telomere DNA efficiently, leading to the activation of ATR, a sensor kinase monitoring ssDNA generated by replication stalling. Recently, it was reported that Timeless, a protein that protects the replication fork, associates with TRF1 and TRF2, thereby facilitating *in vitro* telomere replication in *Xenopus* egg extracts.^11^ It is possible that the overexpressed TRF1 and TRF2 sequestered replication proteins, such as Timeless, from the replication fork, leading to the inefficient replication at telomeres in the above mentioned experiment.^7^–^12^

## Telomeres as a Fragile Site

When cells are exposed to mild replication stresses, such as treatment with aphidicolin, which inhibits DNA polymerases, chromosome breaks are frequently induced. They are typically observed as gaps and constrictions of metaphase chromosomes, and the vulnerable loci are called common fragile site (CFS).^13^ Although the exact locations of CFSs vary between different cell types, and depends on the type of replication stresses, all healthy individuals show CFSs, suggesting that a CFS is an intrinsic characteristic of specific chromosomal regions. Although it appears that the mechanistic details differ among different CFS loci, it is proposed that inefficient replication caused by, for example, a paucity of regional replication origins and a higher-ordered structure of chromatin, underlies the genetic instability associated with CFSs.

Importantly, TRF1-deleted MEF (mouse embryonic fibroblast) cells showed frequent replication fork stalling at telomere repeat DNAs and the adjacent subtelomere DNAs.^10^ Treatment of TRF1-proficinet human cells with low-dose aphidicolin resulted in an increased frequency of morphologically abnormal telomeres in telomere FISH analysis of metaphase chromosome samples, suggesting that telomeres comprise a fragile site. Importantly, the phenotype was observed in TRF1-deficient cells at similar levels in cells with or without aphidicolin application. The TRF1 deletion also produced an increased number of 53BP1-positive telomeres (telomere dysfunction-induced foci, TIFs, Fig. 1a), a hallmark of DNA damage response (DDR) at telomeres caused by telomere protection defects. Taken together, it was concluded that telomeres are a form of CFS. TRF1 plays a pivotal role in protecting telomeres from expressing the fragility.^10^

## Mechanisms of Causing Telomere Fragility

A number of studies mostly relying on *in vitro* experiments have suggested that the GC-rich telomere repeat DNA adopts unusual higher-ordered DNA conformations. Specifically, it is well established that the telomere repeat G-strand DNA forms four-stranded DNA (G-quartet or G-quadruplex, Fig. 1B). Structural analyses revealed that G-quartet is formed by base stackings between consecutive guanine bases within a strand and non-Watson-Crick hydrogen bond-based pairing among the four strands (Hoogsteen base pairing, Fig. 1B). The four strands participating in the formation of a G-quartet can be derived from a single G-rich ssDNA or distinct G-rich ssDNAs (intra-molecular and inter-molecular G-quartets, respectively). A G-quartet is very stable compared to conventional Watson-Crick base-pairing-based double-stranded DNA, and would constitute an obvious thermodynamic obstacle to an advancing replication form. Recently, it has been suggested that G-quartet indeed exists *in vivo*, and possibly has biological relevance, using anti-G-quartet antibodies.^14^

A minimum requirement for a DNA sequence to form an intra-molecular G-quartet is that it contains at least four tandem stretches of G-rich tracts. Each repeat typically contains at least three consecutive guanine nucleotides. The hinge regions connecting the neighboring G-rich tracts may contain several non-G nucleotides. *In silico* analyses indicate that G-rich tracts that potentially form G-quartets are not restricted to telomere repeat DNAs, nor distributed randomly in the human genome. Notably, the G-quartet candidate sequences are overrepresented in pro-proliferative genes, including proto-oncogenes *c-myc*, *VEGF*, *HIF-1α*, *bcl-2* and *c-kit*, especially in the promoter regions, and are scarce in anti-proliferative genes including tumor suppressor genes.^15^–^16^ It has been recognized that G-quartet candidate sequences are frequently found in 5'UTR, and in some cases modulate the translation efficiency of the cognate transcripts.^17^ Other regions that were reported to be rich in the G-quartet candidate sequences include G-rich microsatellites and mini-satellites, rDNA genes, the vicinity of transcription factor binding sites, and regions that frequently undergo DNA double-strand break (DSB) in mitotic and meiotic cell divisions.

Genetic studies indicate that G-rich tracts at telomeres and extra-telomeric regions are regulated by the same pathway. The ion-sulfur-containing DNA helicases comprise a subfamily of helicases, consisting of XPD (xeroderma pigmentosum complementation group D), FANCJ (Fanconi anemia complementation group J), DDX11 (DEAD/H [Asp-Glu-Ala-Asp/His] box helicase 11) and RTEL1 (regulator of telomere length 1). *RTEL1* was identified as a mouse gene essential for telomere maintenance.^18^ Mice homozygously deleted for *RTEL1* were embryonic lethal, and *RTEL1*-deficient ES cells showed short telomeres with abnormal karyotypes. TmPyP4 (meso-tetra[N-methyl-4-pyridyl]porphyrin) is a compound that binds to and stabilizes G-quartet structure. It was found that telomeres were more frequently lost in TmPyP4-treated *RTEL1*-deficient cells compared to untreated cells, suggesting that RTEL1 facilitates telomere DNA replication. Given that RTEL1 is a helicase, it is likely that RTEL1 resolves G-quartet structures at telomeres, thereby enhancing the telomere DNA replication. Interestingly, when *Caenorhabditis elegans* DOG-1, a helicase protein related to FANCJ protein, was inactivated, G-quartet candidate sequences were extensively deleted from the genome.^19^ These results suggest that the ion-sulfur-containing DNA helicases play a role in protecting G-rich sequences from deletion, presumably by inhibiting the DNA replication defects at the G-rich sequences. Taken together, these helicases may ensure the replication of G-rich sequences that frequently harbor regulatory cis-elements and the transcription start sites, and telomere DNAs. Under replication stress, defects in the helicases may lead to chromosomal rearrangements throughout the whole genome.

## Telomerase

Due to the inability for the conventional DNA polymerases to completely replicate linear DNAs, telomere DNA becomes shortened every time cells divide. This phenomenon is called the end replication problem. Specifically, the problem is caused by the difficulty for DNA polymerase α/primase complex to initiate RNA primer synthesis at the very end of linear DNA templates. The G-strand and C-strand of telomere DNAs are invariably replicated by leading strand synthesis and lagging strand synthesis, respectively. Therefore, telomere DNA shortening happens when the C-strand is to be synthesized for the most distal 5′-end.

Progressive telomere shortening due to the end replication problem is most frequently circumvented by a specialized reverse transcriptase, called telomerase, in cells that proliferate indefinitely such as germ cells. Telomerase is active in approximately 90% of clinical primary tumors, whereas normal human somatic cells show negligible telomerase activity in most cases. It was expected that any means to inactivate the telomerase-mediated telomere elongation would provide an ideal anti-cancer therapy that specifically acts on cancer cells.^20^ When telomeres in normal cells are shortened to a threshold level that is minimally required for telomere functions, cells stop dividing due to an active process called replicative senescence. Replicative senescence is supposed to be an effective anti-oncogenic mechanism because it sequesters the genetically unstable cells into an irreversibly arrested state.^21^ However, as the number of non-proliferating cells purged by replicative senescence is increased, the chance that a small number of senescent cells will acquire mutations that bypass the senescence pathway is accordingly increased.^22^ Such cells are produced by accidental and rare mutations that inactivate p53 and/or Rb, two tumor suppressor proteins required for the replicative senescence. The resultant mutant cells resume proliferation until the telomere is indeed inactivated. At this stage, the telomere-dysfunctional cells undergo apoptosis. However, additional mutations and/or epigenetic changes activate telomerase activity in such cells, which re-acquire the ability to elongate telomeres, thereby counteracting the end replication problem, and resulting in uncontrolled proliferation.

Telomerase is a specialized reverse transcriptase. It is an RNA-protein complex consisting of several subunits. Among them, telomerase reverse transcriptase (TERT) and telomerase RNA (TER, encoded by the *TERC* gene) are two components essential for the activity. While *TERC* is ubiquitously expressed, *TERT* is expressed only in telomerase-active cells. Therefore, TERT expression determines whether cells possess telomerase activity. Initially it was thought that telomerase only plays a role in elongating telomeres, but it is now known that it provides telomere-independent functions such as regulating the Wnt signaling pathway and the production of non-coding RNA.^23^–^24^

At the initial stage of investigation, it was thought that telomerase inhibitors would be useful to inhibit tumor growth by depriving cancer cells of the limitless capacity of proliferation.^20^ Because telomerase is a reverse transcriptase, compounds that inhibit reverse transcriptases of viral origins were investigated as telomerase inhibitors with limited success. The template region of the telomerase RNA provides an accessible target to inhibit telomerase. GRN1631 was developed as an anti-sense oligonucleotide that targets telomerase RNA. It has shown some promise in Phase I trials.^25^ As telomerase inhibitors supposedly achieve anti-tumor effects through reducing the telomere length in cancer cells, it was expected that it would take some time before the clinical benefit was realized after administration of drugs.

Since the discovery of *Tetrahymena* telomerase in the 1980s and human telomerase in the 1990s, we now know much about the biogenesis and reaction mechanisms of the enzyme. In particular, it is important to understand how telomerase RNA (TER) is synthesized, matured and incorporated into the ribonuleoprotein (RNP) complex, telomerase.^26^ Mature human TER (hTR) is 451 nt in length. The precursor of hTR contains two hairpin-loops at its 3′-end, a characteristic secondary structure shared by a group of RNAs called H/ACA RNAs. H/ACA RNPs function as enzymes to catalyze the site-specific peusdouridylation of rRNA and small nuclear RNA.^27^ A trimeric protein complex consisting of NHP2, NOP10 and dyskerin are required for processing and maturation of the H/ACA RNAs. Similarly, the trimeric complex processes hTR to yield the mature form of hTR. This maturation step of hTR takes place in the intra-nuclear structure called the Cajal body. By contrast, TERT protein is accumulated in nucleoli. TERT and hTR form the telomerase complex when Cajal bodies are moved to the nucleolar periphery in S phase. As such, TER processing factors including dyskerin (encoded by *DKC1*) are required for the production of the functional telomerase. In the following sections, human diseases that are characterized by impaired production of telomerase will be discussed.

## Telomere Syndrome

Telomere syndrome refers to a spectrum of diseases caused by impaired telomerase activities.^28^ The pathologies grouped in this category have been traditionally diagnosed as two different conditions, namely idiopathic pulmonary fibrosis and dyskeratosis congenita, which will be briefly discussed below.

### Idiopathic pulmonary fibrosis

Idiopathic pulmonary fibrosis (IPF) represents a subset of lung diseases resulting in fibrosis of alveolar interstitium. The prognosis of IPF is poor; approximately 50% of patients die within 3 years after diagnosis.^29^ It has been proposed that IPF occurs when genetically susceptible individuals are exposed to environmental stresses, such as cigarette smoking, bleomycin, asbestos and radiation exposure.^29^ Approximately 2% of the IPF patients are presented as familial cases, suggesting the involvement of genetic background in IPF. The hereditary form is autosomal dominant with variable penetrance. It was found that mutations in telomerase-related genes (*TERT*, *TERC* and *DKC1*) are responsible for the diseases in 15% of familial cases.^30^ The telomere length is excessively shortened in such cases, as expected. Interestingly, it has been reported that telomeres in circulating blood cells are shortened in many sporadic as well as familial cases, despite the fact that there are no mutations in *TERT*, *TERC* or *DKC1*.^31^ The correlation between the telomere length and the occurrence of IPF suggests the causative role of shortened telomeres in IPF.

### Dyskeratosis congenita

Dyskeratosis congenita (DKC) is a hereditary disease characterized by a triad of mucocutaneous symptoms (skin reticulation, dystrophic nails and oral leukoplakia). Dyskeratosis congenita patients frequently develop pulmonary fibrosis, bone marrow failure, and myelodysplasia, which comprise the common causes of death. The diseases are heterogeneous, caused by various mutations in several genes. It was found that X-linked DKC, a severe form of the disease, is caused by mutations in the *DKC1* gene.^32^ In contrast, heterozygous mutations in *TERT* or *TERC* genes underlie the genetic defects in the autosomal dominant form, a rare but clinically mild subtype of the disease.^33^–^34^ In both cases, it is accepted that the reduced telomere length in tissue stem cells leads to the failure of cell renewal of hematopoietic stem cells.

Mutations in *TERT*, *TERC* and *DKC1* cause either IPF or DKC, and some patients show clinical manifestations intermediately between the two diseases. Therefore, it is reasonable to view these diseases as a spectrum of pathology produced by defective telomerase activity. It is notable that malignancies frequently affect IPF and DKC patients (lung adenocarcinoma and myelodysplastic syndrome/leukemia, respectively). Therefore, symptoms displayed by telomere syndrome patients are related to stem cell failure and genetic instability caused by excessive telomere shortening. Intriguingly, autosomal-dominant DKC patients show anticipation, that is, symptoms of a disease are manifested at earlier ages in later generations of one affected pedigree. This can be explained by the fact that patients of later generations possess progressively shortened telomeres.^35^

## C-strand Fill-in Reaction

**Figure 3:**
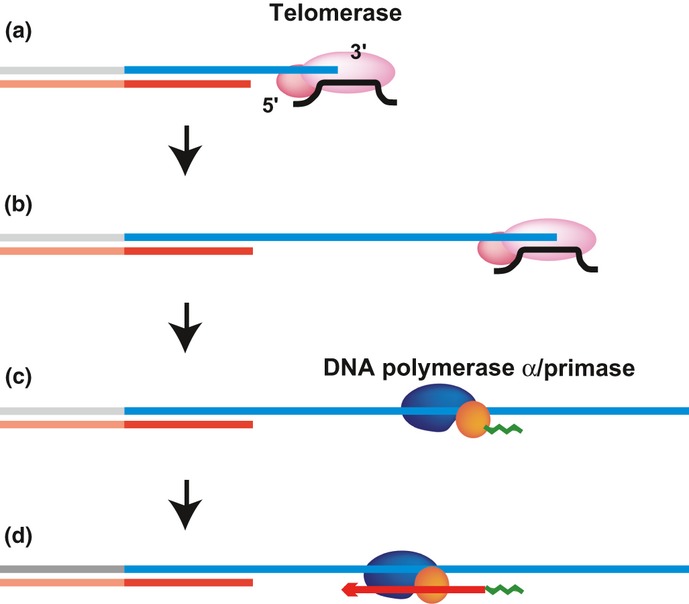
C-strand fill-in reaction. Telomerase leaves a long G-rich strand (a and b). DNA polymerase α/primase complex is supposed to catalyze the fill-in reaction of the C strand DNA. Unlike replication-coupled lagging strand synthesis by DNA polymerase α/primase complex, the enzyme initiates *de novo* RNA primer synthesis followed by DNA elongation (c and d). Wavy green lines and red arrowed lines indicate RNA primers and nascent DNA strands, respectively.

Telomerase elongates only the G-strand but neglects the C-strand. Accordingly, it is necessary to fill-in the C-strand after the G-strand extension by telomerase. Although the precise molecular mechanism remains unknown, it is thought that the C-strand fill-in reaction is achieved by the DNA polymerase α/primase complex. The C-strand fill-in reaction is unique in that the DNA synthesis is not coupled with a replication fork. Instead, it requires de novo RNA primer synthesis followed by DNA synthesis extended by DNA polymerase α (Fig. 3).

Recently, a novel trimeric ssDNA-binding protein complex has been reported in humans.^36^ The Ctc1-Stn1-Ten1 (CST) complex was independently isolated as a protein complex stimulating DNA polymerase α/ primase.^37^ Moreover, it was found that CST complex not only stimulates semi-conservative DNA replication, but mediates the coupled reaction of primer synthesis and templated DNA synthesis in *Xenopus* egg extracts, a finding consistent with the prediction mentioned above.^38^ Interestingly, mutations in the Ctc1 gene are responsible for the hereditary Coats plus syndrome, which is characterized by phenotypes that partly overlap with DKC. Although the molecular mechanisms that leads to clinical manifestations in Coats plus syndrome is not known, these results suggest that additional target genes may be implicated in systemic diseases caused by telomere dysfunction.

## Conclusion

DNA replication at telomeres relies on seemingly telomere-specific molecular pathways. However, it appears that similar pathways also play a role in DNA metabolism involving other genomic regions. Results obtained by telomere biology will contribute to our understanding of how genome-wide chromosome anomalies are produced.
